# Special Issue “Neuropharmacology of Plant Extracts and Their Active Compounds”

**DOI:** 10.3390/ph19030345

**Published:** 2026-02-24

**Authors:** Angel Josabad Alonso-Castro

**Affiliations:** Departamento de Farmacia, Universidad de Guanajuato, Noria Alta, Colonia Noria Alta Guanajuato, Guanajuato 36250, Mexico; angeljosabad@ugto.mx; Tel.: +52-473-732-0006

According to the World Health Organization, 14.2% of the world’s adult population in 2021 suffered from a mental disorder, which is defined an impairment affecting cognition, emotional regulation, or behavior [1]. There are around 200 types of mental disorders. Many people, including friends and family, exhibit discrimination and may lack empathy for patients with mental disorders. The most common mental disorders include anxiety, depression, and bipolar disorder [1]. Attention to mental disorders is a major challenge for many governments. The health care costs and other indirect costs for treating anxiety and depression amount to around 1 trillion dollars each year in the global economy. Despite this, government expenditures for promoting mental health are still insufficient, with low-income countries spending less than 0.04 US dollars per inhabitant to promote mental health [1]. Current antidepressant agents (e.g., selective serotonin reuptake inhibitors and serotonin and norepinephrine reuptake inhibitors) induce side effects such as sexual dysfunction, insomnia, fatigue, weight gain, constipation, and anxiety, among others [2], and some patients treated with these drugs show full remission. Due to the limited efficacy of many psychiatric drugs, in some cases, and the low expenditures of local governments on promoting mental health, many people seek alternative therapies. After the COVID-19 pandemic, the consumption of medicinal plants for treating symptoms associated with anxiety and depression quadrupled in Mexico [3]. This information indicates the need for pharmacological and chemical experiments on the medicinal plants being used to treat mental disorders.

Five of the nine regular articles published in this Special Issue focus on finding possible treatments for acute depression with bioactive compounds from medicinal plants, and all these articles corroborate the importance of continuing to search for new antidepressant drugs. Most of the medicinal plants [*Thunbergia alata* Bojer ex Sims (Acanthaceae), *Argemone platyceras* Link & Otto (Papaveraceae), *Argemone ochroleuca* Sweet (Papaveraceae), and *Centella asiatica* (L.) Urb. (Apiaceae)] from the articles in this Special Issue had previously known ethnomedicinal information regarding their use for treating mental disorders, and the neuropharmacological studies corroborated the ethnomedicinal information. Such evidence indicates the need to continue performing ethnobotanical studies. For instance, it is notable that an ethnobotanical study performed by our research group in rural communities in Guanajuato, a state located in Central Mexico, found that the population refers to the terms of depression or anxiety instead of referring to cultural affiliations as “nervios” (nervousness), “susto,” or “espanto” [4], with the last two denoting sudden fear or a disconnection of the soul from the body. The ethnomedicinal information in many books and sources needs to be updated to consider current mental disorders. Additionally, ethnobotanical studies can incorporate information about other medicinal plants that are used for treating mental disorders. Researchers still need to study the validation of the ethnomedicinal properties of many medicinal plants used in folk medicine for mental disorders.

Two Argemone species (*Argemone platyceras* and *Argemone ochroeluca*) were studied in this Special Issue. The Argemone genus, distributed in the American continent, is a rich source of alkaloids in seeds and flowers. This genus encompasses between 30 and 35 members. The plants from this genus usually bloom all year, which is an advantage when performing phytochemical studies. The pharmacological effects of many of these compounds have not been studied. Alkaloids obtained from the plants of this genus can be a topic of further phytochemical and pharmacological research.

There are articles in this Special Issue that report the neuropharmacological actions of some bioactive compounds. For instance, dihydrosanguinarine (0.1–10 mg/kg p.o.), an alkaloid obtained from *Argemone ochroeluca*, showed antidepressant-like effects in the tail suspension test in mice in acute assays. Hyperoside, a glycosyl flavonol, was the major compound identified in the ethyl acetate extract of *Thunbergia alata*. The antidepressant-like effects of hyperoside in murine models have been previously reported [5]. Tetrahydrocolumbamine (synonym (-)-isocorypalmine) was one of the alkaloids found in an acid methanol extract of *Argemone platyceras* leaves and stems, but the antidepressant-like effects of tetrahydrocolumbamine have not yet been evaluated. Further research is necessary with this compound since the plant extract showed antidepressant-like actions [6]. (-)-syringaresinol, a lignan glycoside, inhibited (IC_50_ = 0.25 ± 0.01 μM) both 4-(4-(dimethylamino) phenyl)-1-methylpyridinium uptake and 4-(4-(dimethylamino) styryl)-*N*-methylpyridinium binding (IC_50_ = 0.96 ± 0.02 μM) by the human serotonin transporter. Furthermore, (-)-syringaresinol (5–20 mg/kg i.p.) also showed antidepressant-like effects in the chronic unpredictable mild stress (CUMS)-induced mouse model by decreasing the immobility time in the forced swimming test and the tail suspension test [7]. Methyleugenol (25–100 mg/kg i.p.), a phenylpropanoid, showed antidepressant-like effects in murine models with the possible participation of adrenergic and dopaminergic systems [8]. The role of microbiota in the development of mental disorders is a novel topic that can be combined with medicinal plants that have neuropharmacological effects and the probiotic compounds derived from plants [9]. Additional research is needed to explore the potential benefits of the combination of probiotics and plants, as well as their active compounds, in treating mental disorders by acting through the modulation of neurotransmitters, receptors, and enzymes. The anti-inflammatory and antioxidant effects of many medicinal plants can also be used to assess their ability to protect against mental disorders. Further studies should also consider the use of other depressant-like models such as the novelty-suppressed feeding test and the sucrose preference test, alongside the tail suspension test and the forced swimming test, with this last test raising questions about the reliability of real despair behavior in rodents [10]. Our research group recently incorporated another parameter (time spent eating) into the antidepressant-like actions of n-butylidenephthalide (50–200 mg/kg p.o.), an isobenzofuranone isolated from many medicinal plants, in an acute assay using the novelty-suppressed feeding test. Other depression-like models should be incorporated due to the limited number of behavioral assays evaluating depression-like behavior in rodents.

In summary, this Special Issue emphasizes the need to perform chronic assays with plant extracts and their active compounds and to perform neuropharmacological evaluations with compounds such as tetrahydrocolumbamine. Chronic toxicological studies are also necessary for medicinal plants and their active compounds before performing clinical trials to guarantee the safe use of these natural products. Pharmacokinetic studies will be important in determining how many times per day a compound should be consumed. Finally, identifying the molecular mechanism of action can facilitate the combination of plant compounds that have different mechanisms of action, allowing for the study of potential plant-derived compound combinations that reduce the dosage of each compound and decrease the possibility of side effects.

## Data Availability

Not applicable.
